# Outcome effects of antiretroviral drug combinations in HIV-positive patients with chemotherapy for lymphoma: a retrospective analysis

**DOI:** 10.1007/s11096-018-0620-1

**Published:** 2018-06-12

**Authors:** F. Sombogaard, E. J. F. Franssen, W. E. Terpstra, E. D. Kerver, G. E. L. van den Berk, M. Crul

**Affiliations:** 1 Department of Clinical Pharmacy, Onze Lieve Vrouwe Gasthuis Hospital, Oosterpark 9, 1090 HM Amsterdam, The Netherlands; 2 Department of Internal Medicine – Oncology and Hematology, Onze Lieve Vrouwe Gasthuis Hospital, Amsterdam, The Netherlands; 3 Department of Internal Medicine – Infection Diseases, Onze Lieve Vrouwe Gasthuis Hospital, Amsterdam, The Netherlands; 4 Department of Clinical Pharmacology and Pharmacy, VU University Medical Center, Amsterdam, The Netherlands

**Keywords:** cART, Chemotherapy, Drug–drug interactions, HIV, Lymphoma

## Abstract

*Background* The combination of combined active antiretroviral therapy (cART) with chemotherapy in the treatment of lymphoma in human immunodeficiency virus (HIV)-positive patients has improved the overall survival of these patients. However, drug–drug interactions between antineoplastic agents and the antiretroviral agents non-nucleoside reverse transcriptase inhibitors (NNRTIs) and protease inhibitors (PIs) can occur by influencing the activity of the CYP3A4 enzyme. So far, little is known about the clinical relevance of this interaction: the effect on the efficacy and toxicity of the chemotherapy. Also, there is no general consensus which cART is preferable in combination with antineoplastic drugs. *Objective* To compare PI-based with NNRTI-based cART on the efficacy and toxicity of chemotherapy in lymphoma patients. *Setting* The Onze Lieve Vrouwe Gasthuis, located in Amsterdam, The Netherlands. *Method* A retrospective observational cohort study including all patients with HIV and lymphoma over a 10-year period. Clinical outcome (response to chemotherapy and survival) and toxicity of chemotherapy (renal, hepatic and bone marrow toxicity as well as dose reduction, treatment delay and discontinuation) was compared in patients with PI based and NNRTI-based cART. Main outcome measure: Response to chemotherapy and survival. *Results* Patients using PI-based cART (n = 22) had a significantly lower 1 year survival compared to NNRTI-based cART (n = 21). No significant differences were observed in reaching complete remission after chemotherapy. No overall significant differences in toxicity and discontinuation of the chemotherapy were observed. However, there was a trend towards more severe bone-marrow toxicity in patients with PI-based cART. In addition, patients with PI-based cART received earlier dose-reduction and treatment delay, indicating increased toxicity in PI-treated patients. *Conclusion* This retrospective study shows that PI-based cART is inferior in combination with chemotherapy to NNRTI-based cART: a lower 1 year survival is observed and dose-reduction and treatment delay occur earlier, possibly based on an earlier onset of toxicity.

## Impacts on practice

- When combining chemotherapy for lymphoma with antiretroviral therapy for HIV, non PI-based drug regimens are preferred.
- Combining PI-based cART with chemotherapy for lymphoma results in a lower 1-year survival rate when compared to NNRTI-based cART combinations.

## Introduction

Combined Active Antiretroviral Therapy (cART) has led to an increased life expectancy of patients diagnosed with human immunodeficiency virus (HIV). Partly because of that, an increase in the incidence of malignancies such as lymphoma is also observed in this group of patients [1, 2]. Previous studies have shown that the combination of cART with chemotherapy increased overall survival of these patients [3–5]. However, even in well-treated HIV-positive patients, the results of lymphoma treatment are, although improving, still not as good as in HIV-negative patients [6–8]. Apart from the HIV infection itself, drug–drug interactions between cART and cytostatics may play an important role in the treatment outcome of patients. Thus far, knowledge about the influence of cART on the efficacy and toxicity of chemotherapy is limited [9, 10].

The antiretroviral drug classes non-nucleoside reverse transcriptase inhibitors (NNRTIs) induce CYP3A4 while protease inhibitors (PIs) inhibit CYP3A4 [9]. Cyclophosphamide, doxorubicin, vincristine and vinblastine are included in the chemotherapy combination schedules used in most lymphoma patients and are mainly metabolized by CYP3A4. A few clinical studies have addressed drug–drug interactions between PIs and NNRTIs and the antineoplastic agents. Two early trials showed the feasibility of combining chemotherapy with cART, although a relatively high percentage of patients experienced severe anemia and neurotoxicy [11] or required granulocyte colony stimulating factor (G-CSF) support while on lymphoma chemotherapy [12]. In addition, reduced cyclophosphamide clearance [13] and increased vinblastine exposure has been described in small patient cohorts [14, 15].

Thus far no up-front dose adjustments have been recommended for HIV-infected patients treated with both antineoplastic agents and cART, even though altered pharmacokinetics and high incidences of myelotoxicity and neurotoxicity have been observed in several independent studies. Especially severe neutropenia in patients receiving concomitant PIs with chemotherapy including vinblastine [16] or cyclophosphamide and doxorubicin [17, 18] has been described. Another study showed an overall doubling in the occurrence of side effects in patients with PI containing cART and chemotherapy compared to patients on cART regimens without PIs [19]. Finally, an increase in neurotoxicity of vinca-alkaloids in patients receiving the PIs ritonavir or lopinavir has been described in patients with Hodgkin lymphoma [20].

The question is whether these drug–drug interactions are clinically relevant and which kind of cART can be best combined to chemotherapy.

### Aim of the study

To investigate whether there are differences in treatment outcome, survival and toxicity of chemotherapy in the treatment of lymphoma between HIV-positive patients with PI-based and NNRTI-based cART.

### Ethics approval

The study was approved by the local ethics committee of Onze Lieve Vrouwe Gasthuis Hospital (OLVG), Amsterdam, The Netherlands and complied with the Declaration of Helsinki.

## Method

This is a retrospective observational cohort study of HIV-positive patients in the OLVG Hospital in Amsterdam who were diagnosed with lymphoma between 2002 and 2012 and were treated with chemotherapy. The treatment outcome of chemotherapy (complete remission or persistent lymphoma) and the survival up to 1 year after finishing chemotherapy were measured in these patients. Toxicity was measured by clinical laboratory chemistry and divided into renal (serum creatinine clearance calculated according to ‘modification of diet in renal disease’ (MDRD) and Cockcroft & Gault), hepatic (alkaline phosphatise (ALP), lactate dehydrogenase (LDH), alanine transaminase (ALT), aspartate transaminase (AST), bilirubin) and myelotoxicity (hemoglobin, leukocytes, platelets, neutrophils) and longitudinally measured during the chemotherapy. Toxicity was scored according to the Common Terminology Criteria for Adverse Events (CTCAE), version 4.03 [21]. Mild to moderate toxicity (grade 1 and 2) and severe to life-threatening toxicity (grade 3 and 4) were grouped.

Parameters used for clinical toxicity were dose reduction of chemotherapy, treatment delay to a later date or discontinuation of chemotherapy. Dose and timepoints of (start of) administrations of the chemotherapy were compared to the standard chemotherapeutic protocols. Discontinuation was defined as the number of cycles received being less than that intended at onset of therapy. Statistical tests were performed with SPSS (Statistical Package for the Social Sciences, SPSS Inc., Chicago, Illinois, USA). A *p* value of 0.05 was considered statistically significant. To compare patient characteristics, Chi squared tests (ordinal data) and Mann–Whitney U tests (continuous data) were used. To compare outcome measures, Chi squared tests (ordinal data) and Log-rank tests (time to reduction or dose delay) were used. To compare survival between groups, a Chi squared test was performed.

## Results

**Fig. 1:**
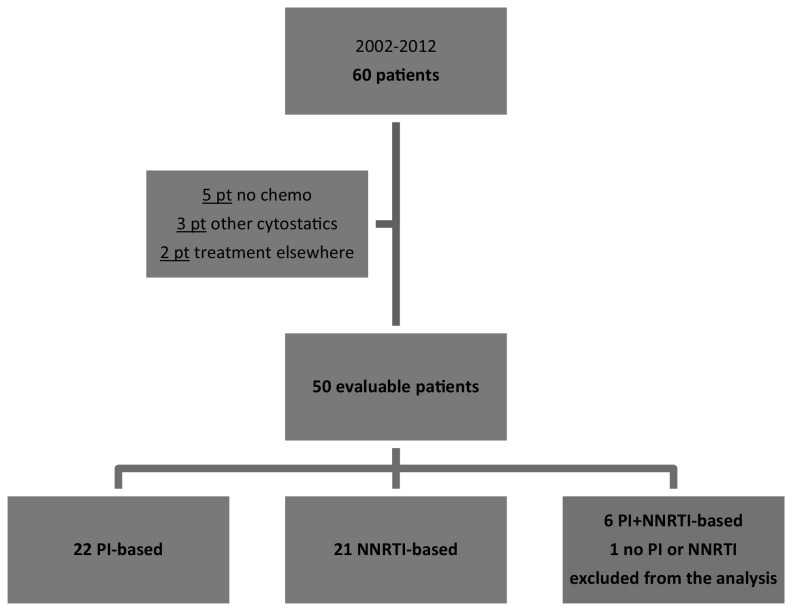
Inclusion of HIV-positive patients diagnosed with lymphoma

From 2002 to 2012, 60 HIV-positive patients have been diagnosed with lymphoma in the OLVG Hospital and were included in the study. Of those patients, 5 had not started with chemotherapy, 3 patients had received chemotherapy without antineoplastic drugs with potential CYP interactions, and 2 patients had received chemotherapy elsewhere. Of the 50 investigated patients, 22 patients were on PI-based cART, 21 on NNRTI-based, 6 patients had cART with both a PI and a NNRTI, and 1 patient was given none of these anti-HIV agents (Fig. 1). The patient characteristics for the PI-based and NNRTI-based cART groups are displayed in Table 1. The 6 patients who received a PI and a NNRTI were excluded of the analyses.

**Table 1:** Characteristics of patients with PI-based and NNRTI-based cART

	PI (n = 22)	NNRTI (n = 21)	*p* value
Male, n (%)	20 (91)	21 (100)	0.16*
Weight (kg), median [range]	74 [52–93]	76 [50–111]	0.57^$^
Length (cm), median [range]	178 [152–190]	180 [166–195]	0.37^$^
Age (years), median [range]	49 [38–72]	47 [35–70]	0.45^$^
Lymphoma, n (%)			
Non-Hodgkin	11 (50)	10 (48)	0.96*
Hodgkin	4 (18)	4 (19)	
Burkitt	4 (18)	3 (14)	
Castleman	3 (14)	4 (19)	
Stage, n (%)			
I	1 (5)	1 (5)	0.48*
II	5 (23)	2 (10)	
III	4 (18)	6 (29)	
IV	4 (18)	7 (33)	
n/a	8 (36)	5 (24)	
Chemotherapy, n (%)			
(R-)CHOP	12 (55)	10 (48)	0.92*
ABVD	5 (23)	5 (24)	
LMBA	4 (18)	4 (19)	
Other	1 (5)	2 (10)	
HIV-1, n (%)	21 (95)	21 (100)	0.32*

*Chi squared test, ^$^Mann–Whitney U test

Table 2 shows treatment outcome and toxicity from laboratory tests and therapy data for both groups. Survival was significantly lower in patients who used PI-based cART compared with NNRTI-based cART (41% mortality vs. 14%, *p* < 0.01). No significant differences in direct treatment outcome were observed between PI- and NNRTI-based cART: in both groups 7 patients achieved complete remission (32% vs. 33%, p = 0.82).

**Table 2:** Treatment outcome, toxicity and adjustments of chemotherapy in the treatment of lymphoma of patients with PI-based and NNRTI-based cART

	PI-based	NNRTI-based	*p* value
*Number of patients*	22	21	
*Clinical outcome, n (%)*			
Complete remission	7 (32)	7 (33)	0.82*
Persistent lymphoma	14 (64)	12 (57)	
Not available	1 (5)	2 (10)	
Survival after 1 year	13 (59%)	18 (86%)	< 0.01*
*Toxicity, n (%)*			
Renal			
Grade 1 + 2	1 (5)	4 (19)	0.14*
Grade 3 + 4	0 (0)	0 (0)	
Hepatic			
Grade 1 + 2	5 (23)	5 (24)	0.99*
Grade 3 + 4	7 (32)	7 (33)	
Bone marrow			
Grade 1 + 2	5 (23)	12 (57)	0.065*
Grade 3 + 4	12 (55)	7 (33)	
*Chemotherapy*			
Dose reduction, n (%)	8 (36)	5 (24)	0.37*
Of which cyclophosphamide	1 (13)	1 (20)	
Doxorubicine	3 (38)	2 (40)	
Vinca-alkaloid	7 (88)	4 (80)	
Time to reduction (50% of patients, days)	130 [116–145]^$^	164 [150–177]^$^	< 0.01^#^
(R-)CHOP (days)	155 [146–164]^$^	163 [157–170]^$^	0.062^#^
ABVD (days)	55 [42–69]^$^	165 [149–182]^$^	< 0.01^#^
Treatment delay, n (%)	10 (45)	5 (24)	0.14*
Time to delay (50% of patients, days)	119 [106–132]^$^	151 [138–164]^$^	< 0.01^#^
(R-)CHOP (days)	153 [144–161]^$^	155 [146–164]^$^	0.31^#^
ABVD (days)	51 [40–62]^$^	155 [139–171]^$^	< 0.01^#^
Discontinuation, n (%)	9 (41)	7 (33)	0.61*

*Chi squared test, ^#^Log-rank test; ^$^mean [95% confidence interval]

There was a trend towards more severe to life-threatening myelotoxicity in the PI based cART group (55% vs. 33%, p = 0.065). Between both groups, no significant difference was observed for nephrotoxicity and hepatotoxicity.

**Fig. 2:**
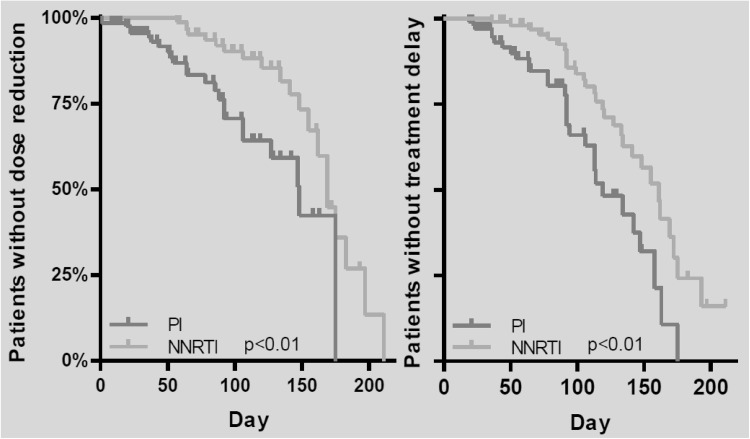
Time to dose reduction and treatment delay of patients with PI-based and NNRTI-based cART

Although no significant difference was observed in the incidence of dose reduction and treatment delay during the entire chemotherapy, the time to dose reduction and/or treatment delay was significantly shorter in the PI-based cART group (130 vs. 164 days, *p* < 0.01; 119 vs. 151 days, *p* < 0.01, respectively, Fig. 2). These effects were more distinct in ABVD (doxorubicin, bleomycin, vinblastine, dacarbazine) regimens compared to (R-)CHOP (rituximab, cyclophosphamide, doxorubicin, vincristine, prednisolone) regimens. Dose reduction of one or more antineoplastic agents was performed three times earlier in the course of therapy in patients using PI-based cART and delay of chemotherapy also occurred three times earlier in the course of therapy in these patients. The vinca-alkaloids vinblastine and vincristine (88% of the patients on PI-based cART and in 80% of the patients on NNRTI-based cART respectively) were the chemotherapy agents for which dose reductions were most often applied.

## Discussion

This study suggests that PI-based cART is inferior to NNRTI-based cART in HIV-patients treated with chemotherapy. Patients using PI-based cART during chemotherapy have a lower 1-year survival compared with NNRTI-based cART, although treatment outcomes after finishing chemotherapy showed no significant difference in the achievement of complete remission. At onset of the chemotherapy, the two groups did not significantly differ from each other in the prognosis and staging of lymphoma, as well as in the choice of chemotherapeutic regimens (see Table 1).

No significant differences were found in laboratory toxicity, possibly due to the size of the cohort. However, there was a trend towards more myelotoxicity in the PI-based cART group. Common toxicity such as sensory and motor neuropathy, nausea and vomiting, alopecia and anorexia were difficult to quantify in this retrospective study. Therefore other parameters were used as surrogates for chemotherapy toxicity, i.e. dose reduction, treatment delay, discontinuation and the timespan of first occurrence in any of these parameters (see Fig. 2; Table 2). In these parameters, no overall significant differences were observed. However, in patients using PI-based cART the time to first dose reduction and treatment delay was significantly shorter. The earlier necessity for dose reductions and treatment delays suggests that severe toxicity occurred earlier in the course of therapy in the PI-based cART group.

A possible explanation for our findings is the occurrence of the drug–drug interactions between chemotherapy and antiretrovirals agents. PI’s inhibit CYP3A4, which are important in the metabolism of the investigated cytotoxic drugs. By inhibiting this enzyme, the exposure to the toxic parent substance and/or metabolites will increase, depending on the specific metabolic pathway of the antineoplastic agent. Decreased metabolism of chemotherapeutic drugs, resulting in increased drug exposure may have affected the occurrence of toxicity caused by antineoplastic agents, as observed by the earlier dose reduction and treatment delay in our study. CYP3A4, although a likely candidate, can as yet not be considered as the unique enzyme involved in the interactions with antiretrovirals: in fact, there are more molecules that would merit further investigation and research in the future (e.g. glycoprotein P, CYP2B6, CYP2C19). It should be noted that both dose reduction and treatment delay are not considered to be the most optimal therapy, because HIV-positive patients should be given the same intensity and treatment of the chemotherapy as HIV-negative patients to achieve the same therapeutic response and overall survival [22]. However, in our study the lesser dose-intensity did not result in lower response rates at the end of chemotherapy. To our knowledge, our study is the first to examine the effects of different cART regiments to dose-intensity of administered chemotherapy and correlating these data with survival and toxicity. Some limitations should be taken into account when interpreting the results. Firstly, the study was retrospective, single center and encompassed a limited number of patients. The assigned chemotherapy regimens however are in line with the general treatment guidelines in lymphoma [23]. Secondly, although no significant differences in baseline characteristics between the two groups were found, we did not take the international prognostic index (IPI) into account as a predictor of clinical outcome for the diffuse large B-cell lymphoma, as this was not recorded routinely during the treatment period of our cohort. Also, responses were recorded as complete response or persistant lymphoma, without subcategorization in partial response, stable disease or progressive disease. Thirdly, we did not study the effect of the chemotherapy on the safety and efficacy of the cART regimen by for example assessing HIV viral load or CD4 counts. It is possible that the patients on PI regimens had failed a previous NNRTI containing regimen and had a further advanced HIV status. Therefore, it cannot be ruled out that the worse survival of our patients with PI-based cART was the result of lack of HIV control rather than the oncological disease per se.

Our findings are in agreement with an early clinical study into the combination of cART and chemotherapy on a cohort of 46 lymphoma patients who were treated with a different regimen (CDE, cyclophosphamide, doxorubicin, etoposide) from ours. Significantly lower neutrophil counts and significantly more infections requiring hospitalization were observed in patients with PIs as compared to patients treated without PIs [17]. From this cohort, no survival data are published. Another study on a relatively small cohort of 32 HIV-positive lymphoma patients also identified PIs (ritonavir and lopinavir) as risk factors for grade 3–4 hematologic toxicity and neuropathy in Hodgkins disease [20]. In this trial, survival data showed no difference when patients were stratified upon clinical or laboratory baseline features or the occurrence of toxicity. In contrast to our study no analysis of survival between the different cART subgroups was performed. A third study including 34 patients treated with CHOP while on cART failed to identify significant differences between PI and non-PI-based cART, neither in efficacy of the chemotherapy nor in its toxicity profile [24]. This difference could be a result of a difference in baseline stage/IPI score between the PI and non-PI group in this trial. The largest published cohort study included 154 patients with any type of cancer, and noted a significant increase in chemotherapy side-effects in patients with PIs when compared to NNRTIs or integrase inhibitors (IIs) [19]. The main goal of this study was to assess the efficacy and safety of the antiretroviral regimen in patients who had cancer, and hence no response rates of chemotherapy were reported. However as in our study, overall survival also indicated worse outcomes for PI treated patients.

Thus far, there is no clear answer to the question to which extent PIs and NNRTIs affect the pharmacokinetics of antineoplastic agents. One study suggests significantly higher vinblastin exposure in patients treated concomitantly with PIs (n = 3) [14], whereas another trial failed to show a significant pharmacokinetic effect of PIs on doxorubicin pharmacokinetics (n = 19) [25]. Larger pharmacokinetic studies as well as prospective trials in this population are necessary to further elucidate the toxicity profiles of chemotherapy in these patients.

Altered pharmacokinetics due to the use of these antiretrovirals agents may result in dose reduction recommendations at the start of the chemotherapy. However, as the effect of CYP interactions may show a large interpatient variability, it is as yet not possible to provide dose adjustment guidelines. Another possibility is to apply cART without drug–drug interactions with the used antineoplastic drugs. In theory, integrase inhibitors (IIs) are eligible while these agents do not interfere with the CYP enzyme system and therefore no alternations in pharmacokinetics and exposure to toxic metabolites are expected. This approach has recently been suggested by a German as well as by an American HIV-treatment group and is supported by a report on a small cohort of Spanish HIV-positive patients undergoing safe and effective chemotherapy while on raltegravir based cART [26–28]. A prospective study of the use of II’s versus NNRTI`s or PI`s is highly recommended.

## Conclusion

This study provides further insight in the effect of different cART regimens on chemotherapy treatment and survival of HIV-positive lymphoma patients. Protease inhibitors have a negative effect on outcome, and should be avoided as much as possible in HIV-patients with cancer.
